# Early and accurate diagnosis of pancreatic cancer?

**DOI:** 10.18632/oncotarget.13142

**Published:** 2016-11-05

**Authors:** Jeremy Sharib, Kimberly Kirkwood

**Affiliations:** Department of Surgery, University of California San Francisco, San Francisco, CA, USA

**Keywords:** IPMN, pancreatic cancer, imaging

Pancreatic cysts are incidentally identified in up to 40% of all abdominal imaging studies in adults performed for reasons unrelated to the pancreas, but only a small percentage of these cysts harbor malignancy. Among the potentially malignant pancreatic cysts, Intraductal Papillary Mucinous Neoplasms (IPMN) represent the vast majority, and their incidence is increasing. IPMNs accounted for <5% of resected pancreatic cystic neoplasms prior to 1990, but, with the sharp rise in use of abdominal imaging for diagnosis and surveillance, as well as improvements in imaging resolution, IPMNs are now the indication for over half of resected pancreatic cysts [1]. This change, termed a “man-made epidemic,” highlights the clinical importance of understanding the natural history of pancreatic cysts.

IPMNs are classified as either “main duct,” derived from the major pancreatic duct that drains digestive fluid into the duodenum (MD-IPMN), or “branch duct,” arising from one of its tributaries (BD-IPMN). Since about 50% of MD-IPMNs contain foci of either high grade dysplasia or invasive cancer, and resection of early stage cancers confers a survival advantage, current recommendations are to resect all MD-IPMNs in patients who are suitable surgical candidates. In contrast, only 15-20% of BD-IPMNs harbor or will develop malignancy, while the remainder are indolent, and observation alone is adequate. Accurate identification of patients with non-metastatic malignant BD-IPMN is important since resection is often curative, whereas once the cancer has spread to the liver, few patients survive 5 years. Thus the objective is to identify and resect all BD-IPMNs with either high grade dysplasia or invasive cancer, while avoiding unnecessary surgery for benign lesions.

The increasing detection of IPMNs has been accompanied by a parallel rise in the number of patients adversely impacted by “overtreatment” of benign pancreatic cysts, meaning unnecessary surgical removal of indolent lesions. In one recent study 77% of resected BD-IPMNs were benign [2]. Pancreatic resection carries a mortality rate of 2-4%, and 30-50% of patients will suffer complications from surgery and a prolonged recovery. One must weigh the risks of surgery against recent evidence that only 4% of patients with BD-IPMN developed cancer after four years surveillance.

Improvements in technology have led us to our current landscape, in which we can detect a lesion that has a very small chance of harboring pancreatic cancer with high sensitivity. While surgery offers a cure, the operation is so burdensome it is best reserved for patients who actually have high grade dysplasia or early invasive cancer. These considerations create difficult decisions for clinicians and patients who endeavor to weigh the morbidity and mortality of resection against the uncertain biology of their pancreatic cysts. In the absence of accurate diagnostic tests, consensus guidelines have been developed to aid clinicians in decision making, however, several studies show that these criteria inaccurately identify lesions with high grade dysplasia, relying heavily on crude imaging characteristics such as lesion size which has limited predictive value.

The study by Hanania et al [3], published in this issue seeks to bridge this diagnostic gap by applying quantitative imaging “radiomics” to improve the predictive power of axial CT to identify high grade dysplasia in IPMN. The authors analyzed numerous raw CT data fields to create a complex algorithm that assigned quantitative variables that differentiated tumors based on degree of dysplasia. This predictive model was then applied to images from patients with previously resected lesions. In this cohort, 36% of patients had unnecessary resections based on low grade dysplasia on histology. Hanania's radiomic model improved the sensitivity and specificity to predict high grade and invasive cancer in this cohort to 97 and 88 percent respectively.

Hanania shows that quantitative radiomics can improve classification of the degree of dysplasia in IPMN. Moreover, the radiomic signature also detects genomic patterns that may further differentiate benign and aggressive tumors [4]. In a second study in this issue, Permuth et al [5] found that quantitative imaging could be used to identify genomic expression patterns in malignant IPMNs. These efforts demonstrate the potential for radiomics to not only predict high grade dysplasia, but also to build an imaging library that might reflect the genomic signature of newly diagnosed lesions, and thereby obviate the need for invasive biopsy or cyst fluid collection. Recent investigation has shown predictive power of genetic mutations [6] and proteomics [7] in classifying IPMN. While current techniques to analyze proteomic activity are cumbersome, these 2 studies offer the potential to relate specific genomic changes to quantifiable imaging variables. These studies also open the pipeline to applications, including functional radiomic techniques, to provide highly specific information about tumor biology. Importantly, such radiomic evaluation fits within the current diagnostic paradigm, and therefore has the potential to greatly improve diagnostic accuracy with minimal additional cost.

The escalating use of sensitive abdominal imaging modalities is creating an epidemic of pancreatic cystic lesions, the majority of which are benign IPMNs. These studies suggest that quantitative radiomics has the potential to improve the power of noninvasive current cross-sectional imaging techniques to accurately classify these lesions and provide information about their biology. Once validated, diagnostic platforms should be standardized to enable consistent data acquisition and analysis across institutions. Multicenter evaluation of large cohorts of patients with pancreatic cysts are needed to refine current evidence-based clinical guidelines, with the anticipated benefit of both enhancing early detection of curable pancreatic cancers as well as reducing the burden of unnecessary major surgical procedures.
